# A Fast TaqMan^®^ Real-Time PCR Assay for the Detection of Mitochondrial DNA Haplotypes in a Wolf Population

**DOI:** 10.3390/genes16080897

**Published:** 2025-07-28

**Authors:** Rita Lorenzini, Lorenzo Attili, Martina De Crescenzo, Antonella Pizzarelli

**Affiliations:** 1 Istituto Zooprofilattico Sperimentale del Lazio e della Toscana M. Aleandri, 58100 Grosseto, Italy; rita.lorenzini@izslt.it (R.L.); martina.decra99@gmail.com (M.D.C.); antonellapizzarelli@gmail.com (A.P.); 2 Dipartimento di Scienze della Terra dell’Ambiente e della Vita (DISTAV), Università di Genova, 16132 Genoa, Italy

**Keywords:** *Canis lupus*, wolf, dog, hybrid, genetics, mtDNA, Italy, conservation, management, diagnostics

## Abstract

**Background/Objectives**: The gene pool of the Apennine wolf is affected by admixture with domestic variants due to anthropogenic hybridisation with dogs. Genetic monitoring at the population level involves assessing the extent of admixture in single individuals, ranging from pure wolves to recent hybrids or wolf backcrosses, through the analysis of nuclear and mitochondrial DNA (mtDNA) markers. Although individually non-diagnostic, mtDNA is nevertheless essential for completing the final diagnosis of genetic admixture. Typically, the identification of wolf mtDNA haplotypes is carried out via sequencing of coding genes and non-coding DNA stretches. Our objective was to develop a fast real-time PCR assay to detect the mtDNA haplotypes that occur exclusively in the Apennine wolf population, as a valuable alternative to the demanding sequence-based typing. **Methods**: We validated a qualitative duplex real-time PCR that exploits the combined presence of diagnostic point mutations in two mtDNA segments, the *NDH-4* gene and the control region, and is performed in a single-tube step through TaqMan-MGB chemistry. The aim was to detect mtDNA multi-fragment haplotypes that are exclusive to the Apennine wolf, bypassing sequencing. **Results**: Basic validation of 149 field samples, consisting of pure Apennine wolves, dogs, wolf × dog hybrids, and Dinaric wolves, showed that the assay is highly specific and sensitive, with genomic DNA amounts as low as 10^−5^ ng still producing positive results. It also proved high repeatability and reproducibility, thereby enabling reliable high-throughput testing. **Conclusions**: The results indicate that the assay presented here provides a valuable alternative method to the time- and cost-consuming sequencing procedure to reliably diagnose the maternal lineage of the still-threatened Apennine wolf, and it covers a wide range of applications, from scientific research to conservation, diagnostics, and forensics.

## 1. Introduction

The Apennine wolf is regarded as a distinct ancient subspecies (*Canis lupus italicus*, Altobello, 1921) showing morphological, genetic, and genomic uniqueness [1]. After centuries of population isolation and decline, verging on extinction in the 1970s, the wolf is currently experiencing a natural, steady recovery throughout its entire peninsular distribution area and in the Alps [1,2].

In early 2025, the European Commission submitted a proposal to downgrade the protection status of the wolf from “strictly protected” to “protected” [3]. However, the species is not yet out of the woods in Italy, and there are concerns for its survival in the long run. Population sizes and dynamics are highly affected by anthropogenic mortality, through direct and indirect persecution [4]. Furthermore, hybridisation with domestic dogs poses a further worrying, though often underestimated, threat to the long-term conservation of the wild gene pool [5,6]. Evaluating the extent of hybridisation is, therefore, crucial for monitoring the phenomenon at the population level [6,7] and plays a key role in supporting effective conservation efforts.

Assessing the genetic status of a putative Apennine wolf (i.e., pure wolf, recent hybrid, or backcross) involves a demanding diagnostic procedure based on a Bayesian assignment of multilocus genotypes from a panel of autosomal short tandem repeat (STR) loci, as well as the analysis of uniparental markers, i.e., STR haplotypes on the Y chromosome [8,9] and mitochondrial DNA (mtDNA) haplotypes [10].

Currently, the diagnostic identification of mtDNA haplotypes in the wolf is carried out via Sanger sequencing of coding and control region (*CR*) variants [10,11,12,13]. However, the sequencing process, while relatively easy to perform, is time- and cost-consuming, even when short segments are examined. On the other hand, a complete diagnostic DNA-based procedure for unknown putative Apennine wolf samples does require assessing the subspecific mitochondrial lineage. Furthermore, when wolf × dog hybrids are identified, it is also desirable to confirm that hybridisation occurred unidirectionally, i.e., between a domestic male dog and a female wolf, as is observed in nature, with a few rare exceptions [14]. In this scenario, all hybrids should theoretically carry wild mtDNA haplotypes.

Wolf populations across Europe bear different, and sometimes exclusive, composite mtDNA haplotypes, consisting of a combination of point mutations in the *NDH-4* (*ND4*), *ATP6*, and *COX3* genes, as well as the *CR* [12,13]. The Apennine wolf carries two diagnostic multi-fragment haplotypes (WH14 and WH19 after [12]) that can be distinguished by a unique association of two single-nucleotide polymorphisms (SNPs) at the *ND4* gene and one SNP at the *CR*. In particular, the variants at two SNP sites of *ND4* are fixed in the N5 haplotype [13], while the variants at one SNP site of the *CR* define the W14 and W16 haplotypes [12,13]. The combination of N5 with W14 or W16 yields the two unique multi-fragment mtDNA haplotypes WH14 and WH19.

In this study, we developed a fast, reliable, and affordable single-tube duplex real-time PCR method to detect WH14 and WH19 mtDNA haplotypes that are exclusive to the Apennine wolf population. The assay exploits the combined presence of specific point mutations in the *ND4* and *CR* segments through TaqMan^®^-MGB chemistry for high-throughput testing. This method represents a valuable alternative to sequencing while requiring only basic expertise and unsophisticated laboratory equipment. Following our diagnostic procedure, sequencing is bypassed in the first instance and left, if necessary, only to those samples that show unexpected or unclear results, for individual haplotype identification.

## 2. Materials and Methods

### 2.1. Samples

We tested our real-time PCR assay using a total of 149 samples, collected from 2020 to 2024. They comprised 38 reference pure wolves from the Apennine population, 21 Dinaric wolves from Slovenia, 52 medium- and large-sized dogs (25 mongrels from central Italy and 27 purebreds, including wolf-like breeds: Maremma Sheepdog, Bracco Italiano, Argentine Dogo, Belgian Shepherd, English Setter, Border Collie, Golden Retriever, Czechoslovakian Wolfdog, German Shepherd, Corso Dog, Drahthaar, Pit Bull, Siberian Husky), and 33 Apennine wolf × dog hybrids (introgressed wolves and recent hybrids) from peninsular Italy. A total of 5 additional known non-Apennine wolf × dog hybrids were also included in the analyses.

Pure wolves and hybrids had previously been identified by genetic analyses and a Bayesian assignment procedure, on the basis of a panel of 23 nuclear STR loci on autosomes and 5 STR loci on the Y chromosome, following a validated protocol [9], and were selected from our in-house tissue biobank. Wolf and hybrid samples consisted of muscle tissue from individuals that had died of natural causes, poaching, or accidentally, mainly due to poison bait targeted at other species or collisions with vehicles. Found-dead wolves were recovered by local authorities and delivered to Istituto Zooprofilattico Sperimentale di Lazio e Toscana for diagnostic or forensic purposes. No animals were captured and/or sacrificed specifically for the purposes of this study, nor were any laboratory experiments or investigations conducted on live animals. No ethical approval was necessary to use samples from found-dead animals. Dog samples were muscle or salivary swabs. The latter were collected from owned dogs with the permission of their owners, while tissue samples were gathered exclusively from dogs that had died from disease or an accident, or because they were victims of animal crimes under investigation by the authorities. Samples of non-Apennine wolf × dog hybrids were salivary swabs obtained from animals that had been seized from illegal dog breeders by the authorities in Italy. Samples were stored at −20 °C until processing.

### 2.2. DNA Extraction, Primer and Probe Design

DNA was isolated from approximately 25 mg of muscle or one salivary swab using the QIAamp DNA Mini Kit (Qiagen, Hilden, Germany), following the manufacturer’s instructions. DNA quantification was obtained with the QuantiFlor^®^ dsDNA system (Promega, Madison, WI, USA).

We developed a qualitative duplex real-time PCR using primers and TaqMan probes targeting two diagnostic sites in the mitochondrial *CR* and *ND4* gene of *Canis lupus*. To set up the assay we aligned 50 wolf and dog sequences for each target, selected from our in-house database of mtDNA sequences, as well as some downloaded from the GenBank online repository (Table S1), and manually designed two sets of primers/probes (Table 1). Using the canine mitogenome NC_002008 as a reference, the assay was designed on positions 11515 (A>G) in the *ND4* gene and 15654 (C>T) in the *CR*.

**Figure 1:**
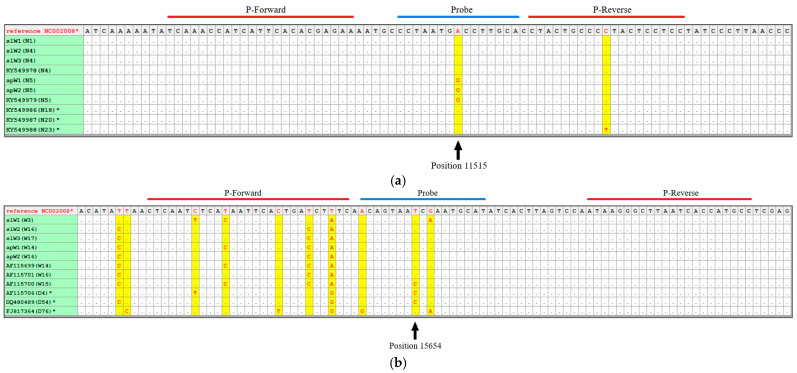
The alignment of the sequences used (partial list) to design primers and probes for a duplex real-time PCR assay at two variable sites in the mitochondrial DNA of *Canis lupus*: (**a**) *ND4* gene; (**b**) *CR*. apW = Apennine wolves and slW = Slovenian wolves used in this study. The accession numbers for the sequences downloaded from GenBank are provided. * = dog sequence; N1–N23 = *ND4* haplotypes; W3–W17, D4–D76 = *CR* haplotypes; reference NC002008* = dog reference mitogenome sequence.

According to the literature [12,13] and our alignments (Figure 1), nucleotide G at the 11515 site in *ND4*, producing a non-synonymous amino acidic variation Thr vs. Ala, is exclusive to the N5 haplotype. This haplotype, in combination with W14 or W16 at the *CR*, yields the two unique multi-fragment mtDNA haplotypes that are exclusive to the Apennine wolf population (WH14 and WH19, following [12]) (Table 2). Conversely, when the N5 haplotype is combined with W15 at the *CR*, a different composite haplotype is produced (WH17), which is found in wolves from Greece. In dogs and wolves from other populations (Greece, Bulgaria, and Slovenia, amongst others) nucleotide A is alternatively present at the 11515 site, yielding, for example, the widespread multi-fragment WH18 when it combines with W16 at the *CR* (Table 2). The site at position 15654 in the *CR* (Figure 1) presents nucleotide T in the great majority of dogs and wolves, including those from the Apennine population (haplotypes W14 and W16) (Table 2). In the Greek wolves carrying the W15 haplotype and in some purebred dogs and mongrels, a C residue is otherwise present.

The combination of the G variant at the diagnostic SNP 11515 at *ND4* and the T variant at the diagnostic SNP 15654 in the *CR* is typical of the two exclusive haplotypes of the Apennine wolf (multi-fragments WH14 and WH19), whereas it is absent in any other dog or wolf population across Europe. In our TaqMan real-time PCR assay, VIC- and 6FAM-labeled probes specifically anneal when a diagnostic G is present at *ND4* (haplotype N5) and T is concurrently present in the *CR* (Table 1). Therefore, haplotypes WH14 and WH19 are only detected if the amplification curves from both probes are displayed. In all other cases, only one curve, or none, are expected.

The guide of the Primer Express software 3.0.1 (Applied Biosystems) was followed to search for the best in silico primer location, efficiency of binding, and melting temperatures for our primers/probe sets (Table 1). The sequences of the designed primers were checked for specificity of binding in the expected genes of *C. lupus* through the BLAST algorithm (https://blast.ncbi.nlm.nih.gov/Blast.cgi, accessed on 5 January 2025), with positive results.

### 2.3. Real-Time PCR Conditions

Before optimising a duplex real-time PCR assay, we checked for positive amplification products in five Apennine wolves by running singleplex end-point and real-time PCRs based on the newly designed primers. End-point PCR reactions contained 2.5 μL of 10X Gold buffer (Applied Biosystems), 0.2 mM of each dNTP, 2.5 mM MgCl_2_, 0.4 μM of primers, 1U of AmpliTaqGold polymerase (Applied Biosystems), and 3 µL template DNA, reaching a final volume of 25 μL. Amplifications were performed in a Veriti thermal cycler (Applied Biosystems, Foster City, CA, USA) and consisted of an initial 5 min denaturation step at 95 °C, 33 cycles of 30 s at 95 °C, 30 s at 55 °C, and a 2 min extension step at 72 °C, followed by 5 min at 72 °C. The conditions for singleplex real-time PCR differed from those of the duplex version (see below), except for primer concentration, which was 900 nM for both pairs.

The optimised duplex real-time PCR reactions contained 5 µL TaqMan GTXpressTM Master Mix (Applied Biosystems), 700 nM *CR* primers, 900 nM *ND4* primers, 200 nM probes (FAM/MGB- and VIC/MGB-fluorophores TaqMan^®^ Probes by Applied Biosystems, Life Technologies), and an amount of genomic DNA in the recommended range (see Results), resulting in a total volume of 10 µL. The amplification profiles consisted of a hold stage of 20 s at 95 °C and a PCR stage of 40 cycles at 95 °C for 15 s and 60 °C for 1 min, with a final post-read stage of 60 s at 25 °C. Thermal cycling was performed in a QuantStudio 7Flex (Applied Biosystems, Life Technologies). The default settings of the Applied Biosystems software analysis system were initially used to automatically calculate the threshold cycle (C_t_) values. Two negative controls (reagents with no DNA template) were included in each amplification run, one from the DNA extraction room and one from the PCR mix set-up room. The presence of an amplification curve produced by annealing the 6FAM-labeled TaqMan probe simultaneously served as an internal positive control and diagnostic amplification for the multi-fragment WH14 and WH19 haplotypes, as it excludes the *CR* W15 haplotype (see Table 2 and Figure 1).

### 2.4. Validation Studies

Basic validation studies were performed on our qualitative duplex real-time PCR assay to define its limitations in detecting the specific target DNA and providing reliable and reproducible results. The assessment of specificity, defined as the property of a method to respond exclusively to the characteristic of interest [15], was the first step in the validation process. In our case, specificity is provided by the generation of two amplification curves (VIC and 6FAM) exclusive to the Apennine wolf. To verify that, we used genomic DNA from all samples (N = 149) and tested them at the two selected variable mtDNA sites through our new developed primers/probe sets. The same samples were used as independent replicates to assess the reliability of the assay. A positive control (DNA target from Apennine wolf) and negative controls (no DNA template in the reactions) were included in each run to monitor amplification success and the lack of contamination, respectively. As an ancillary step to the assessment of specificity, we sequenced 15 Apennine wolves, 15 Apennine wolf × dog hybrids, 21 Slovenian wolves, and 5 dogs using primers from previous studies (L-Pro/H576 for *CR* [10]; For11093/Rev11741 for *ND4* [16]) to verify the expected haplotype sequences with respect to the real-time PCR assay results.

Once specificity was assessed, sensitivity, repeatability, and reproducibility were tested using five Apennine wolf samples as independent replicates. Seven ten-fold DNA serial dilutions (from 10 ng to 10^−5^ ng) were set up for each replicate to determine the limit of detection, LOD, defined as the lowest amount of template DNA that yields a positive signal in at least 95% of total PCR reactions [15], as well as the variation in the performance of different replicates [17]. In order to assess the reproducibility of the results, the same tests were repeated twice by two operators on different days.

Validation tests were undertaken under the recommendations of the Scientific Working Group for Wildlife Forensic Sciences [18] and the Animal, Plant and Soil Traces (APST) expert working group of the European Network of Forensic Science Institutes [19].

## 3. Results

### 3.1. Evaluating Primers/Probe Sets

Singleplex end-point PCRs in five Apennine wolves yielded one 68 bp long amplicon using the primers designed on the *CR*, and one 80 bp long amplicon using the primers for the amplification in the *ND4* gene. No extra PCR products were observed. As a second step, we successfully verified that both sets of primers/probes produced positive and clear results in singleplex real-time PCRs through the visualisation of the expected amplification curves. Next, we set up a duplex real-time PCR assay as a single reaction and tested for the expected amplification of both products, obtaining positive results in all five samples.

### 3.2. Validation

Once the amplification of the expected targets was verified through clear visualisation of both curves in the first 5 Apennine wolf samples, we applied our assay to the entire set of 149 samples. The results (Table 3) revealed that all reference pure wolves from the Apennine population showed two amplification curves, suggesting that they all carried one of the multi-fragment haplotypes, WH14 or WH19. All wolf × dog hybrids, both old (i.e., introgressed wolves with minimal dog ancestry) and recent (e.g., F1s and F2s), also showed two curves, supporting the hypothesis of unidirectional, preferential maternal-biased hybridisation. Conversely, in the Slovenian wolves, either no curves or only one curve was obtained (from the 6FAM probe binding to the *CR*). Furthermore, in no case were two curves detected in non-Apennine wolf × dog hybrids. Most dogs (90%), including wolflike dogs, showed no amplification curves, including the 6FAM curve, which, theoretically, was expected to occur based on the presence of T at the 15654 SNP of the *CR* that would yield probe annealing. This suggests that the limiting factor for positive amplification in this primer/probe set is the specificity of the primer sequences.

**Figure 2:**
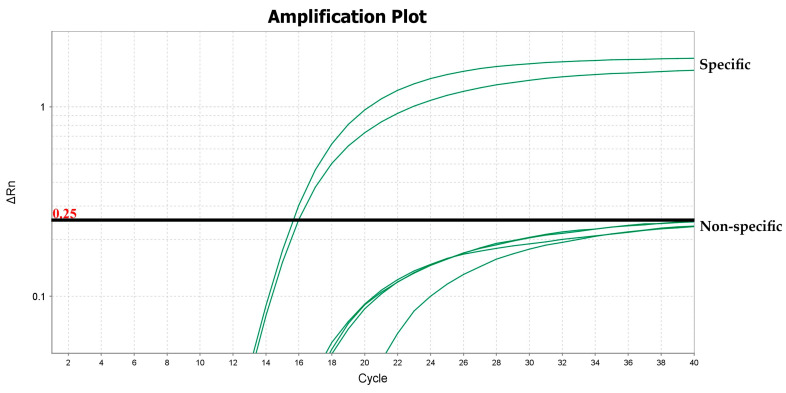
An amplification plot of the mitochondrial *ND4* gene based on the VIC-probe, performed using 10 ng of genomic DNA from Apennine wolves (specific curves) and dogs (non-specific curves).

Only 10% of the dogs (as well as 10% of the Slovenian wolves) exhibited a positive 6FAM curve, suggesting a high binding affinity of the primers to their haplotype sequences. As expected, the primers/VIC-probe set consistently amplified in 100% Apennine wolves and 100% hybrids. However, the probe exhibited non-specific, but clearly less efficient, amplifications in four dog samples. Therefore, to address the presence of non-specific curves, the proper placement of the fluorescent threshold for this probe was determined by manual analysis at 0.25. This adjustment produced a negligible impact on specific amplifications in all Apennine wolves and their hybrids (Figure 2). For the 6FAM curve, we let the threshold value be set automatically (not shown). None of the negative controls, using both automatic and manual thresholds, were scored as positives during either the evaluation of the primers/probe sets or the validation steps.

To evaluate the results of our real-time PCR assay, we verified that the haplotype sequences of the relevant samples were actually those expected according to the literature. Therefore, we sequenced a 500 bp long segment of the *CR* and a 520 bp long segment of the *ND4* gene in 15 Apennine wolves, 15 Apennine wolf × dog hybrids, 21 Slovenian wolves, and 5 dogs (including the 4 individuals exhibiting non-specific VIC-probe curves). The results showed that all Apennine wolves (Table 2) and their hybrids (not shown) carried the expected WH14 and WH19 haplotypes, while 19 Slovenian wolves carried the WH3 haplotype, 1 the WH18 haplotype, and 1 the WH20 haplotype, in full agreement with previous studies [10,12,13]. Meanwhile, 2 dogs showed haplotype DH20 [13], while two new haplotypes were found in the remaining 3 dogs. It should be noted that in no case was the diagnostic SNP present at site 11515 of the *ND4* gene, suggesting the possibility of off-target effects (e.g., amplification of *Numts)* for the VIC-probe. However, these effects were easily minimised by setting an appropriate fluorescence threshold.

The analytic sensitivity of our method was evaluated by using ten-fold DNA serial dilutions (from 10 ng to 10^−5^ ng) of five Apennine wolf samples (replicates). The 4 dogs that showed non-specific VIC-probe curves were also included in the validation step to confirm that the selected threshold was adequate to exclude them as positive samples. The results (Table 4) showed that when using amounts of DNA as low as 10^−5^ ng, the reactions still yielded two amplification curves in wolves, with C_t_ values ranging from 16.23 (SD 0.78) at 10 ng DNA to 39.11 (SD 1.10) at 10^−5^ ng when applying a threshold of 0.25. In dogs, a non-specific VIC curve showed a C_t_ value of 39.80 (SD 0.58) at a DNA amount of 10 ng, while at reduced template DNA contents, the level of fluorescence dropped below the threshold. Consequently, the detection limit, LOD, was set at 10^−5^ ng, and the cut-off point of the assay, defined as the C_t_ value corresponding to the detection limit, was 39.11 (SD 1.10). However, an optimal range of genomic DNA concentration between 1 ng and 10^−4^ ng is recommended for highly reliable results.

The repeatability and reproducibility of the assay were assessed using samples from wolves, with five replicates at different genomic DNA dilutions run twice on different days by two operators. The intra-assay coefficients of variation (CV, Table 4) relative to the optimal DNA concentrations (from 1 ng to 10^−4^ ng) were low, ranging from 4.0% to 7.5%. The inter-assay CV values were in the range of 7.0% and 9.7% (not shown in Table 4). These results indicate that the TaqMan-based duplex real-time PCR developed in this study is a highly repeatable and reproducible assay.

## 4. Discussion

The assessment of mtDNA variability is a milestone in wolf genetics, ranging from phylogeography and divergence times of populations [1,13] to molecular systematics and dispersal patterns [2]. Furthermore, the analysis of maternally inherited mtDNA haplotypes concurs, in combination with bi-parental and paternal genetic markers, to trace the ancestral gene pool of an individual, as well as to identify admixed wolf × dog hybrids and verify the direction of hybridisation [14].

In this study, we developed and validated a qualitative real-time PCR assay to detect the mtDNA haplotypes that occur exclusively in the Apennine wolf population. Based on two diagnostic SNPs in the *ND4* gene and the *CR*, our single-tube duplex real-time PCR assay proved highly reliable, even with DNA concentrations as low as 10^−5^ ng. Using the proper threshold value, the assay reached 100% specificity and high repeatability, meaning that in no case, other than the expected (i.e., Apennine wolves and their hybrids), were two amplification curves obtained in other samples during testing. All dogs, including wolflike dogs, and non-Apennine wolf × dog hybrids displayed either one or no curve, which produced neither false negatives (one or no curve in the Apennine wolf) nor false positives (two curves in non-Apennine wolves and their hybrids).

We also examined samples of Slovenian wolves from the Dinaric range of the species to further validate the specificity of our qualitative one-step real-time PCR. Dinaric wolves show the highest genetic similarity with the Apennine wolf, sharing, for example, the mitochondrial W16 haplotype at the *CR* [13,20]. Hence, they were the best candidates for possible cross-reactivity in our test. Moreover, given their geographical proximity, they can easily cross the Italian north-eastern border and mate with the wolves of the Apennine population [21]. It may, therefore, be necessary to diagnose crosses between wolves from different gene pools. The results of our survey showed that in no case did Slovenian wolves display two amplification curves simultaneously, thus excluding any cross-reactivity of the test. Conversely, in a few cases, they showed only one curve (predictably, the one produced through annealing of the 6FAM-labeled probe at the *CR*). When all the Slovenian wolves were sequenced to identify multi-fragment haplotypes (*ND4* + *CR*, totalling more than 1000 bp), they showed the WH3, WH18, and WH20 haplotypes, in agreement with the literature [12,13,21].

According to our test’s design, failure to display one or both curves would indicate either the absence of the SNP of interest in the amplified segment (in which case the probe does not anneal and fluorescence is not captured) or reduced binding affinity of the primers, up to failure to anneal, due to mutations in the target sequence (in which case the segment of interest does not amplify at all). The latter holds particularly true for the *CR*, where the forward primer specific to the Apennine wolf sequence was designed on a rather variable stretch. Not surprisingly, therefore, only some dogs and Slovenian wolves (actually a few, 10% each) exhibited a 6FAM curve.

The real-time PCR assay developed in this study also showed a very high analytic sensitivity, with a genomic DNA amount as low as 10^−5^ ng still producing positive amplifications. The limit of detection that we suggested for highly reliable results (10^−4^ ng) is in line with other real-time PCR assays targeting mtDNA, e.g., those aimed at quantifying the DNA of domestic species in foodstuff [22,23] or detecting pathogenic parasites in human and animal blood [24].

High sensitivity is a property that makes a test particularly useful in the diagnosis of non-invasive field samples (faeces, hairs, urine, biological traces, decomposed corpses) for scientific or forensic purposes. These samples often contain a low amount of highly degraded DNA, so the use of a sensitive molecular test targeting small mtDNA fragments may be the only opportunity to obtain biological data on the species under study, or evidence in forensic cases. It should be emphasised, however, that an appropriate, more stringent approach to assay validation (i.e., assessment of acceptance criteria for DNA extraction and PCR modules, performance requirements) is required if non-invasive samples are to be used.

Our assay can be applied in scenarios involving degraded DNA traces, for example, to salivary swabs collected around bite wounds in the case of livestock predation [11], or when assaults on humans by canids occur [25,26], in order to rapidly identify whether Apennine wolves or dogs are responsible for the attack.

One of the most attractive features of our real-time PCR diagnostic procedure is the possibility of detecting exclusive mitochondrial haplotypes of the Italian wolf population without resorting to sequencing technology, according to an approach that is both accurate and cost-effective. Sequencing can then be performed only when unexpected or unclear results are obtained, e.g., if a known hybrid yields one amplification curve or none at all instead of the two expected curves, or when the precise identification of single- or multi-fragment haplotypes in either wolves, hybrids, or dogs is required.

The application of the present real-time PCR is not only used to complete the diagnostic DNA-based procedure for the identification of the Apennine wolf, but it also serves to verify the directionality of mating when studying wolf × dog hybridisation. Due to ethological reasons, mating between wolves and dogs is sexually asymmetrical, so that hybrids result from a male dog mating with a female wolf [27]. Theoretically, however, the opposite is also possible, and instances have in fact been observed in the wild, albeit very rarely, of a male wolf mating with a female dog [14]. In this regard, our test can reliably confirm the wild maternal lineage of a hybrid or, if not, be preparatory to sequencing, in order to define the nucleotide composition of the underlying haplotype(s). In our case, all hybrids in the entire dataset of samples analysed through real-time PCR showed wolf maternal lineage, supporting, first, the usual directionality of hybridisation for the Apennine wolf population, and second, the lack of need for sequencing. Last, but not least, the assay is rapid, affordable, and easy to perform. Based on a simple PCR reaction mixture and basic laboratory equipment, neither specific operator expertise nor sophisticated instruments are required to perform the analysis. Following our protocol, a batch of 96 DNA samples loaded into a dedicated 12 × 8-well plate can be processed and the results can be displayed in less than two hours.

## 5. Conclusions

The single-tube duplex real-time PCR developed and validated in this study proved to be a fast, reliable, and accurate method to detect mtDNA haplotypes of the Apennine wolf, a still-endangered, protected subspecies from the Italian peninsula. Based on the detection of two specific point mutations in the *ND4* gene and the *CR*, this procedure does not rely on time- and cost-consuming sequencing analysis to detect the mitochondrial lineage, and it requires only basic expertise of operators and unsophisticated laboratory equipment. Interestingly, the assay covers a range of applications, from completing and increasing the reliability of the current diagnostic procedure for differentiating pure Apennine wolves from dogs and wolves from different gene pools, to evaluating maternal mitochondrial contribution in hybrids. Relying on the amplification of small DNA fragments, the method allows the use of non-invasive field samples, from hairs and faeces to degraded tissues and biological traces. This feature can be highly attractive and valued in both scientific studies and forensic investigations.

## Figures and Tables

**Table 1:** The primers and probes of a duplex real-time PCR assay targeting two variable positions in the mitochondrial *ND4* gene and the *CR* to detect haplotypes that are exclusive to the Apennine wolf population. The diagnostic sites of the TaqMan probes are in italic lowercase. The positions are according to the reference dog sequence NC_002008. F = forward primer, R = reverse primer. Tm = melting temperature; GC = guanine and cytosine residues (from Primer Express v3.0.1, Applied Biosystems).

Target	SNP Position	Primer Sequence (5′ → 3′)	Tm (°C)	GC (%)	TaqMan Probe	Tm (°C)	GC (%)	Amplicon Size (bp)
*ND4*	11515	F-TCAAACCATCATTCACACGAGAA R-GGATAGGAGGAGTAGGGGCAGTAG	59.1 59.5	39 58	VIC-CCTAATGgCCTTGCA-MGB NFQ	65	53	68
*CR*	15654	F-CTCAATCTCACAATTCACTGACCTATC R-GGCATGGTGATTAAGCCCTTAT	58.3 58.0	41 45	6FAM-ACAGTAAtCGAATGCAT-MGB NFQ	66	35	80

**Table 2:** The combined mtDNA haplotypes (fragments *ND4* + *CR*) in the wolf populations from Italy, Greece, Bulgaria, and Slovenia. The data for Greece and Bulgaria are from the literature [12], while the haplotypes of Apennine and Slovenian wolves were obtained in this study. The sample numbers for each combined haplotype are indicated in square brackets. The haplotype nomenclature follows [10,12]. The variable sites are 11515 at the *ND4* gene and 15654 at the *CR*, according to the reference dog sequence NC_002008.

Haplotype			Apennines (Italy)	Greece	Bulgaria	Slovenia
	*ND4*	*CR*				
WH14	N5 (G)	W14 (T)	+ [14]			
WH19	N5 (G)	W16 (T)	+ [1]			
WH17	N5 (G)	W15 (C)		+		
WH18	N4 (A)	W16 (T)		+	+	+ [1]
WH15	N4 (A)	W14 (T)		+		
WH3	N1 (A)	W3 (T)				+ [19]
WH20	N4 (A)	W17 (T)				+ [1]

**Table 3:** The results of applying a duplex real-time PCR assay to the entire sample set of wolves, hybrids, and dogs, using a fluorescence threshold of 0.25 for the VIC-labelled probe. Automatic threshold setting was used for the 6FAM probe. Two curves = the presence of mtDNA haplotypes exclusive to the Apennine wolf; no (one) curve = the presence of other mtDNA haplotypes (see text for details). N = sample size.

	N	No Curve	One Curve	Two Curves
Apennine wolf	38	0	0	38
Apennine wolf × dog hybrid	33	0	0	33
Slovenian wolf	21	19	2 (6FAM)	0
Non-Apennine wolf × dog hybrid	5	5	0	0
Dog	52	47	5 (6FAM)	0

**Table 4:** The Ct values of wolves (specific VIC curves) and dogs (non-specific VIC curves), corresponding to ten-fold dilutions of the target mtDNA template, based on a threshold of 0.25. SD = standard deviation, ND = not determined. The coefficients of variation (CV) were calculated for wolves only.

	Wolf			Dog	
DNA Content (ng)	Mean	SD	CV (%)	Mean	SD
10	16.23	0.78	4.2	39.80	0.58
1	20.11	0.83	4.0	ND	ND
10^−1^	24.31	1.40	5.6	ND	ND
10^−2^	28.85	2.01	6.6	ND	ND
10^−3^	33.23	2.67	7.5	ND	ND
10^−4^	37.40	2.63	6.3	ND	ND
10^−5^	39.11	1.10	2.3	ND	ND

## Data Availability

The original contributions presented in this study are included in the article. Further inquiries can be directed to the corresponding author(s).

## Supplementary Materials

The following supporting information can be downloaded at: https://www.mdpi.com/article/10.3390/genes16080897/s1, Table S1: The accession numbers of online mitochondrial control region (*CR*) and NDH-4 (*ND4*) sequences downloaded from GenBank, used in the alignments to design primers and probes for a duplex real-time PCR assay [10,13,16,28,29,30,31,32].

## Abbreviations

The following abbreviations are used in this manuscript:

*CR*	Control region
CV	Coefficient of variation
MGB	Minor Groove Binder
mtDNA	Mitochondrial DNA
*NDH-4*	NADH dehydrogenase 4
PCR	Polymerase Chain Reaction
SD	Standard deviation
SNP	Single-nucleotide polymorphism
STR	Short tandem repeat
