# Supplementary material for: A Fast TaqMan® Real-Time PCR Assay for the Detection of Mitochondrial DNA Haplotypes in a Wolf Population
Source: Genes (Basel). 2025 Jul 28;16(8):897. doi: 10.3390/genes16080897 (PMC12385737; doi:10.3390/genes16080897)
Supplement: Supplementary file 1 [file genes-16-00897-s001.zip › genes-3780196-supplementary.pdf]

Table S1. Accession numbers of online mitochondrial control region (CR) and NDH-4 (ND4) sequences downloaded from GenBank and used in the alignments to design primers and probes for a duplex real-time PCR assay.

ND4	CR	Reference
KY549975		Montana et al. 2017
KY549976		Montana et al. 2017
KY549977		Montana et al. 2017
KY549978		Montana et al. 2017
KY549979		Montana et al. 2017
KY549980		Montana et al. 2017
KY549981		Montana et al. 2017
KY549982		Montana et al. 2017
KY549983		Montana et al. 2017
KY549984		Montana et al. 2017
KY549985		Montana et al. 2017
KY549986		Montana et al. 2017
KY549987		Montana et al. 2017
KY549988		Montana et al. 2017
KF661045		Thalmann et al. 2013
KF661087		Thalmann et al. 2013
KF661050		Thalmann et al. 2013
KF661080		Thalmann et al. 2013
FJ817363		Baranowska et al. 2009
KU644670		Koblmüller et al. 2016
KU696405		Koblmüller et al. 2016
KU644663		Koblmüller et al. 2016
KU644667		Koblmüller et al. 2016
KU644668		Koblmüller et al. 2016
KF661041	KF661041	Thalmann et al. 2013
KF661047	KF661047	Thalmann et al. 2013
KF661054	KF661054	Thalmann et al. 2013
KF661052	KF661052	Thalmann et al. 2013
KF661052	KF661052	Thalmann et al. 2013
KF661087	KF661087	Thalmann et al. 2013
FJ817358	FJ817358	Baranowska et al. 2009
DQ480499	DQ480499	Bjornerfeldt et al. 2006
DQ480489	DQ480489	Bjornerfeldt et al. 2006
NC002008	NC002008	Kim et al. 1998
	DQ480489	Bjornerfeldt et al. 2006
	FJ817363	Baranowska et al. 2009
	KU696390	Koblmüller et al. 2016
	KU696389	Koblmüller et al. 2016
	KU696388	Koblmüller et al. 2016
	KU696398	Koblmüller et al. 2016
	KU696397	Koblmüller et al. 2016
	FJ817364	Baranowska et al. 2009
	KF661055	Thalmann et al. 2013
	KF661055	Thalmann et al. 2013
	KF661074	Thalmann et al. 2013
	KF661064	Thalmann et al. 2013
	AY656742	Shahid 2004 direct submission

	EU408245	Webb et al. 2009
	AF115706	Randi et al. 2000
	AF115699	Randi et al. 2000
	AF115700	Randi et al. 2000
	AF115701	Randi et al. 2000
	AF338807	Randi et al. 2000
	AF344301	Randi et al. 2000
	AF338812	Randi et al. 2000
	AF344302	Randi et al. 2000
	AF338781	Randi et al. 2000
	AF338772	Randi et al. 2000
	AF338776	Randi et al. 2000
	AF338775	Randi et al. 2000
	AF338777	Randi et al. 2000

References

Kim KS, Lee SE, Jeong HW, Ha JH. The complete nucleotide sequence of the domestic dog (Canis familiaris) mitochondrial genome. Mol Phylogenet Evol. 1998 Oct;10(2):210-20. doi: 10.1006/mpev.1998.0513. PMID: 9878232.

Thalmann O, Shapiro B, Cui P, Schuenemann VJ, Sawyer SK, Greenfield DL, et al. Complete mitochondrial genomes of ancient canids suggest a European origin of domestic dogs. Science. 2013 Nov 15;342(6160):871-4. doi: 10.1126/science.1243650. PMID: 24233726.

Montana, L., Caniglia, R., Galaverni, M., Fabbri, E., Ahmed, A., Bolfíková, B. Č., ... & Randi, E. (2017). Combining phylogenetic and demographic inferences to assess the origin of the genetic diversity in an isolated wolf population. PloS one, 12(5), 1-19.

Baranowska,I., Hultin Jaderlund,K., Nennesmo,I., Holmqvist,E., Heidrich,N., Larsson,N.-G., Andersson,G., Wagner,G.H., Hedhammar,A., Wibom,R. and Andersson,L. 2009. Sensory ataxic neuropathy in golden retriever dogs is caused by a deletion in the mitochondrial tRNATyr gene. PLoS Genet. 5 (5), E1000499

Bjornerfeldt,S., Webster,M.T. and Vila,C.2006.Relaxation of selective constraint on dog mitochondrial DNA following domestication. Genome Res. 16 (8), 990-994

Koblmüller, S., Vilà, C., Lorente-Galdos, B., Dabad, M., Ramirez, O., Marques-Bonet, T., ... & Leonard, J. A. (2016). Whole mitochondrial genomes illuminate ancient intercontinental dispersals of grey wolves (Canis lupus). Journal of Biogeography, 43(9), 1728-1738.

Randi,E., Lucchini,V., Christensen,M.F., Mucci,N., Funk,S.M.,Dolf,G. and Loeschcke,V. 2000. Mitochondrial DNA variability in Italian and East European wolves:detecting the consequences of small population size and hybridization. Conserv. Biol. 14 (2), 464-473

Webb,K.M. and Allard,M.W.2009.Mitochondrial genome DNA analysis of the domestic dog: identifying informative SNPs outside of the control region. J. Forensic Sci. 54 (2), 275-288
